# Department of Therapeutics

**Published:** 1894-01

**Authors:** 

**Affiliations:** Demonstrator of Physiology and Lecturer on the History of Medicine in the Medical Department of the University of Texas, etc.; Galveston


### DEPARTMENT OF THERAPEUTICS.

EDITED BY DAVID CERNA, M. D., PH. D.,
Demonstrator of Physiology and Lecturer on the History of Medicine in the Medical Department of the University of Texas, etc.

THIOFORM.—In the Berlin correspondence (*Medical Press and Circular*, November 1, 1893), reference is made to this recent remedy. *Thioform*, the bismuth salt of dithrosalicylic acid, is almost insoluble in water, and is suitable for surgical dressings, like iodoform, to which, however, it is said to be superior. It has none of the disadvantages of iodoform or its surrogates, bismuth, salicylic acid, boric acid, and the like. It has sufficient disinfecting power to permit wounds to heal under it antiseptically. It is almost non-irritant, it can be blown into the conjunctival sac, and possesses drying properties. It has no smell, is a deodorant, and relieves pain, so that Prof. Hoffman, who has investigated it, can thoroughly recommend it.

---

CREOSOTE IN LATENT TUBERCULOSIS.—Blanchard, of Geneva (*Medical Press and Circular*, November 8, 1893), has published seventeen cases of latent phthisis treated by creosote, in which he not only obtained the improvement so often witnessed in confirmed cases, but a complete and rapid cure. The drug was always administered *per rectum*. Each enema contained five drops of creosote suspended in olive oil, the yolk of an egg, and a half-pint of warm water, and increased gradually to forty drops daily. The patients took no other medicine. The conclusions of the author are as follows: Pulmonary phthisis can be cured at its *début*. The action of the creosote is very effective in the latent form of the malady, and can be relied upon as a curative agent. No inconvenience can occur from its use, even if the diagnosis were not correct.

---

QUININE AS AN OXYTOCIC.—Writing upon the above subject, C. W. Canan, of Virginia (*American Medico-Surgical Bulletin*, December, 1893), expresses explicit belief in the power of quinine as an oxytocic. He has employed the medicament in forty-three cases of natural labor, in thirty-seven of which it produced uterine contraction, increased their power, regulated pains, and aided in hastening a prolonged case of labor. The author formulates the following conclusions: 1. Quinine does not exert its influence directly upon the uterus, but does act indirectly. When inertia is due to depression of the vital forces, quinine, in small doses, becomes a valuable stimulant. 2. When given to promote uterine contraction, it does not produce unnatural and persistent pains that are so often produced by ergot, which endanger both the life of the mother and the child. 3. The same effect can be obtained by administering it in much smaller doses than was formerly given, thereby not causing the patient any unpleasant symptoms. 4. That patients who take quinine as an oxytocic where it is needed, have but little trouble during their parturition.

---

TREATMENT OF NIGHT SWEATS. — S. Bernheim (*Semaine Médicale—American Medical and Surgical Bulletin*, December, 1893) recommends, above all, the following solution for hypodermatic injection against phthisical night sweats:

℞ Salicylic acid....................1 part,
Alcohol ........................3 parts,
Glycerin .......................2 parts,
Distilled water ..................5 parts.

M. Inject 2 c. c. (representing about 20 ctg.—3 grains—of salicylic acid) of this solution at bedtime.

This dose, repeated for four to five consecutive days, is said to generally suffice for combating the most obstinate sweats.

As another serviceable formula, is mentioned the following:

℞ Salicylic acid....................1 part,
Ether ..........................2 parts.

Dissolve, filter through absorbent cotton, replace the quantity of ether eventually evaporated during filtration, and add, in small proportions and with agitation:

Oil of sweet almonds . ........7 parts.

Inject every evening 2 to 4 c. c. (½ to 1 fl. dr.) of this oily solution.

---

ATROPINE NOT EFFICIENT IN A CASE OF MORPHINE POISONING.—The *Therapeutic Gazette* of December, 1893, publishes the details of a case of morphine poisoning, reported by Taylor, in the *Anstralasian Medical Gazette*, in which it is easily seen that atropine was absolutely *worthless* in counteracting the noxious effect of the opiate. Why the report should be entitled "Notes on a case of morphine poisoning *successfully* (italics are ours) treated by atropine," we cannot understand, except by assuming that the prefix "*un*" was inadvertently divorced from the word "successfully" by the carelessness of the type-setter. That the title of the report, as given by the *Therapeutic Gazette*, is misleading, becomes evident from reading carefully said report. The case recovered, but in this result the belladonna alkaloid took apparently no part whatever. This is plainly stated by the reporter himself, who, in one of the concluding paragraphs of his article, says: "The atropine exercised its full physiological action on the heart and iris. The respiration was *not much*, if at all, affected by it, and *had not artificial respiration been resorted to and kept up almost constantly during the whole time, there can be no doubt that a fatal result would have occurred.*" (Italics are ours.)*

---

*Attention has already been called, in an article by the editor of this department, in last January's (1892) issue of the JOURNAL, to the fact that no real antagonism exists between opium and belladonna. The reader is referred to Dr. Cerna's paper.

### NOTES ON DERMATOLOGY.

REPORTED BY ISADORE DYER, M. D., NEW ORLEANS, LA.,
Professor of Dermatology in the New Orleans Polyclinic; Lecturer and Clinical Instructor in Skin Diseases, Medical Department Tulane University, etc.

Part IX. of the International Atlas of Rare Skin Diseases has just appeared. This excellent work, published in Hamburg, by Leopold Voss, consists of a series of articles, with superb illustrations, representing everything modern in the discovery of the rarer forms of skin diseases. The current number contains four original articles, fully illustrated. The first, by V. Babes, is "On a special variety of malignant Pemphigus." The patient lived only seven days after admission to the hospital. "The eruption at first consisted of papules, the center of which was occupied by a lax bulla filled with a clear liquid. The tonsils were covered with a plastic exudation. The scrotum was denuded of epidermis, and covered with granulations and a thin, slimy layer."

The author notes the frequent occurrence of epistaxis as a prominent symptom. "On the thorax and abdomen, there was a disseminated eruption of bullæ, which have here and there collapsed. . . . On the upper and lower extremities, especially on the palms and soles, the epidermis was raised by a serous exudation." Note is made of subsequent crusting, and marked infiltration, especially of the mucous membrane. Pathological examinations discovered very little characteristic change in the spinal cord. The right malphigii was found reduced in thickness, and the lesions seem confined to the epidermis. There was a certain amount of cell proliferation into the corium. The peripheral nerves were found sclerosed, and similar changes were discovered in the smaller arteries.

Concluding the interesting analysis of the case, the author says: "Besides, even in its malignant, gangrenous, diphtheritic, proliferating, or foliaceous varieties, pemphigus does not possess a similar distributión observed in this case, which depends on innervation, and which includes parts not usually affected by pemphigus."

The second case reported is one of "An uncommon form of Keratodermia, or 'Porokeratosis,' " by Vittorio Mibelli.

The third case is exceedingly interesting in many ways:

"Psoriasis conjunctivæ palpebrarum (psoriasis opthalmica)," by Arnold Sack. The patient reported had a general eruption of psoriasis. The face was unaffected, excepting for the patch to be described. "This patch, somewhat semicircular in form and about one centimetre in diameter, occupies exactly the middle of the lower lid of the right eye. . . . The shining, silvery patch presents hardly any point of difference from the other psoriatic elements of the body surface. A faintly re-inflammatory halo encircles it. . . . If the lower lid is everted, the following conditions can be seen: Where the equator of the semicircular lesion is to be sought, a ledge-like elevation is seen along the ciliary border of the whole extent of the diameter of the patch. This goes also around the inner side of the lid. Here the affection seems to occupy an equally large territory on the conjunctiva as on the outer side of the lid." The author submitted this lesion, which was duly excised, to a microscopic examination. The result was entirely satisfactory, and gave evidence of a truly psoriatic patch. This case stands in good evidence of the appearance of psoriasis on the mucous surfaces, a point heretofore *sub judice;* it is a rare specimen of an unusual condition.

The fourth and last article of this part, is written by Pierleone Tommasoli, on "Akrokeratoma hystriciforme hereditarium," which is really a congener of ichthyosis.

In the October number of the *British Journal of Dermatology*, Dr. Leslie Phillips has written a very satisfactory article on "Kristalline." This is the name given to a proprietary liqueur made in this country. It resembles collodion, but is far more transparent, and is capable of carrying in solution many substances refused by collodion. It, in addition, is, more transparent, and does not spoil so quickly. It is suggested as a basis for the local administration of various medicaments used upon the skin.

The *Journal of Cutaneous and Genito-Urinary Diseases* for November, reproduces a paper read by Dr. R. B. Morison, of Baltimore, at the last annual meeting of the American Dermatological Association. The subject of the paper is "Cosmetics." According to Dr. Morison, the use of cosmetics is not necessarily deleterious. He looks upon certain therapeutic measures in the improvement of the complexion, and the treatment of acne is included, as classed with cosmetics. Galvanism in acne, measures to remove the hyperæmia following acne, the treatment of warts, all are considered in this class. We can hardly appreciate such generalization, when the usual acceptance of the term "cosmetics" is as applied to remedies used for conditions not necessarily morbid, as acne certainly is. The article, however, contains many valuable suggestions, and many points were brought out in the discussion by the members. Galvanism was endorsed as beneficial in selected cases of acne; opinion was divided upon the usefulness of electrolysis in hypertrichosis. Stress was laid upon the necessity of attention to the hygiene and general health of the subject afflicted with acne. Little attention was given to the cosmetics of the shops, such as bleaches, and washes, etc. Such are usually condemned, but society demands remedies for the improvement of complexions not necessarily marred by disease, but disfigured by abnormal functions, or temporary conditions. Rough skins, oily skins, pallid cheeks, unmanageable, dry, or oily hair, are some of the things a physician is questioned about, and as he usually discountenances the subject, the patient seeks the charlatan, or the druggist. I was impressed with a prefatory remark made by Dr. Paschkes in his little work on "Cosmetics": . . . "Still the physician is wrong in overlooking the study of the science of cosmetics, for with a knowledge of this, he can not only be of service to patients who suffer from slight blemishes, but he can guard them against dangerous experiments."

